# Are Predictive Energy Expenditure Equations Accurate in Cirrhosis?

**DOI:** 10.3390/nu11020334

**Published:** 2019-02-04

**Authors:** Tannaz Eslamparast, Benjamin Vandermeer, Maitreyi Raman, Leah Gramlich, Vanessa Den Heyer, Dawn Belland, Mang Ma, Puneeta Tandon

**Affiliations:** 1 Department of Medicine, University of Alberta, 130 University Campus, Zeidler ledcor Centre, Edmonton, AB T6G 2X8, Canada; eslampar@ualberta.ca (T.E.); mangma@ualberta.ca (M.M.); 2 Alberta Research Center for Health Evidence, Pediatrics, 4-496 Edmonton Clinic Health Academy, University of Alberta, Edmonton, AB T6G 1C9, Canada; ben.vandermeer@ualberta.ca; 3 Department of Medicine, University of Calgary, 6D26 TRW Building 3280 Hospital drive NW, Calgary, AB T2N 4N1, Canada; mkothand@ucalgary.ca; 4 Department of Medicine, Royal Alexandra Hospital, University of Alberta, Edmonton, AB T5H 3V9, Canada; lg3@ualberta.ca; 5 Alberta Health Services Nutrition Services, University of Alberta Hospital, Edmonton, AB T5H 3V9, Canada; Vanessa.DenHeyer@albertahealthservices.ca (V.D.H.); Dawn.Belland@albertahealthservices.ca (D.B.)

**Keywords:** indirect calorimetry, predictive equations, resting energy expenditure, cirrhosis

## Abstract

Malnutrition is associated with significant morbidity and mortality in cirrhosis. An accurate nutrition prescription is an essential component of care, often estimated using time-efficient predictive equations. Our aim was to compare resting energy expenditure (REE) estimated using predictive equations (predicted REE, pREE) versus REE measured using gold-standard, indirect calorimetry (IC) (measured REE, mREE). We included full-text English language studies in adults with cirrhosis comparing pREE versus mREE. The mean differences across studies were pooled with RevMan 5.3 software. A total of 17 studies (1883 patients) were analyzed. The pooled cohort was comprised of 65% men with a mean age of 53 ± 7 years. Only 45% of predictive equations estimated energy requirements to within 90–110% of mREE using IC. Eighty-three percent of predictive equations underestimated and 28% overestimated energy needs by ±10%. When pooled, the mean difference between the mREE and pREE was lowest for the Harris–Benedict equation, with an underestimation of 54 (95% CI: 30–137) kcal/d. The pooled analysis was associated with significant heterogeneity (I^2^ = 94%). In conclusion, predictive equations calculating REE have limited accuracy in patients with cirrhosis, most commonly underestimating energy requirements and are associated with wide variations in individual comparative data.

## 1. Introduction

Malnutrition is present in up to 80% of patients with decompensated disease [1,2], related to altered energy metabolism [1], malabsorption and reduced oral intake due to anorexia, dysguesia, early satiety and imposed dietary restrictions [2,3]. A robust predictor of pre- and post-liver transplant mortality and morbidity, malnutrition is associated with hepatic decompensation, reduced quality of life and higher rates of infection [4,5,6,7,8].

An optimal supply of macro and micronutrients is the mainstay of nutrition therapy in cirrhosis. Underfeeding and overfeeding are both associated with potential adverse consequences including reduced cardio-respiratory muscle strength, immunosuppression, increased risk of infections (underfeeding) and hyperglycemia, immunosuppression and liver fat infiltration (overfeeding) [9]. To minimize these adverse consequences, practitioners need to be able to accurately determine energy requirements. 

In healthy individuals, the resting energy expenditure (REE) accounts for the majority of the total energy expenditure [10,11]. Clinically, REE can either be *estimated* using predictive equations or *measured* with more precision using direct or indirect calorimetry (IC) [12,13]. To translate the REE into an energy prescription, the mREE and pREE are adjusted by activity and/or stress factors that vary depending upon the clinical status of the patient. 

Although indirect calorimetry carried out using a metabolic cart is the gold standard REE tool [14], practical challenges limit the utility of IC measured resting energy expenditure (mREE) in day-to-day practice [14,15]. These challenges include a lack of routine availability, high cost and the time required to carry it out (at least 30 min) [16]. 

Predictive equations (predicted REE, pREE) such as the Harris–Benedict (HB) and Mifflin equations [17] have been utilized as surrogates for estimating energy expenditure. Although commonly used, there have been concerns raised about the accuracy of these equations in clinical populations [16,18], with estimated energy requirements ±40% the physiological energy needs measured using calorimetry [11,19,20,21] and little consensus on which predictive equation is most applicable for use. Notably, the majority of predictive equations have been developed in healthy subjects, are based on regression analysis of basic demographic data such as weight, height, age, and gender [22,23,24,25] and are unable to account for the changes in metabolic rate associated with acute conditions and modifications in energy expenditure that occur with changes in body composition [26]. 

Recognizing the prevalence and adverse impact of malnutrition and the risks of under and overfeeding, accurate nutrition prescriptions are an essential component of cirrhosis patient care. Using a systematic review of the published literature in patients with liver cirrhosis, the aim of this study was to compare the degree of discrepancy between caloric requirements estimated using predictive equations (predicted REE, pREE) versus indirect or direct calorimetry measurements (measured REE, mREE).

## 2. Materials and Methods 

### 2.1. Search Strategy 

In conjunction with an academic health librarian, we developed a comprehensive search strategy consisting of Medical Subject Heading (MeSH) keywords: “Calorimetry”, “indirect calorimetry”, “direct calorimetry”, “resting energy expenditure”, “resting metabolic rate”, “hypermetabolism”, “Liver cirrhosis”, “end-stage liver disease”, “chronic liver disease”, “Harris-Benedict”, “Mifflin”, “Schofield”, “predictive equation”, as well as combinations of the above terms. The search strategy is found in Table S1. The following databases were searched: Scopus (www.scopus.com) (1966–2017), US National Library of Medicine (PubMed.gov) (restricted to last 180 days to obtain the ‘in process’ citations missed by MEDLINE), MEDLINE (1966–2017) and EMBASE (1988–2017). In some cases, the reference list of the retrieved articles was used to find other relevant studies. The references of reviews were used to pearl additional articles. The original search was carried out in November 2016 and updated in November 2017.

### 2.2. Trial Selection 

Screening of titles and abstracts was performed by two independent reviewers (TE and PT). Full-length versions of selected articles were then retrieved and assessed for inclusion using predefined eligibility criteria: 

Inclusion criteria: Full-text observational or controlled research studies in adult patients (aged >18 years) with cirrhosis defined on the basis of consistent histopathologic, or laboratory, clinical, and ultrasound features, of any sex or nationality, published in the English language. 

Exclusion criteria: Studies were excluded if they were written in a language other than English, were not published as full reports (editorials, opinion papers, review articles, letters to editors and conference abstracts), had >20% of patients with hepatocellular carcinoma (HCC), did not have extractable numerical data reporting mREE/pREE ratios or percentages, or calculated total energy expenditure (TEE) instead of REE.

### 2.3. Data Extraction

The following data were collected: The patient’s hospitalization status (inpatient vs. outpatient), sex, age, weight, height, etiology of liver disease, ascites, presence of HCC, Child–Pugh (CP) grade and score, model for end-stage liver disease (MELD) score, mREE, pREE and the name of the equation or method used to predict REE. Subgroup analyses were planned to analyze the differences in energy expenditure based on sex, type of predictive equation, hospitalization status (inpatient versus outpatient) and study origin, quality rating (low/moderate vs. high risk of bias) and whether dry weight was mentioned as being accounted for (dry vs. no dry weight). 

### 2.4. Outcome Measures

The primary outcome of interest was the discrepancy between the measured (gold standard) versus the calculated energy requirements using predictive equations. The formulas for the predictive equations used in each study are presented in Table S3.

### 2.5. Statistical Analysis 

To estimate pooled effects, mean differences were combined using DerSimonian–Laird random-effects meta-analysis. A negative effect size (pREE ˂ mREE) indicates underestimation, and a positive effect size (pREE ˃ mREE) indicates overestimation. Heterogeneity was tested via the Cochran’s Q test and measured inconsistency by I^2^. The source of heterogeneity was studied by subgroup analyses based on sex, body mass index (BMI), etiology of liver disease, clinically detectable ascites, CP score, as well as MELD score. *p*-values less than 0.05 were considered statistically significant. Statistical analyses were done using SPSS 16.0 (SPSS Inc., Chicago, IL, USA), and the mean differences across studies were pooled with Review Manager 5.3 (The Cochrane Collaboration, Copenhagen, Denmark).

### 2.6. Study Quality and Publication Bias Assessment

Study quality was assessed using the PRISMA statement [27]. Quality assessment and grading was assigned to each article, as described below:

**“Low risk”:** Low risk of bias, indicated that the majority of the research design domains, see Supplementary information Table S2, were appropriately controlled.

**“Moderate risk”:** Insufficient data, indicated that the research design, implementation and analysis was not strong, was not adherent to indirect calorimetry protocols or had errors that would risk measurement accuracy.

**“High risk”:** High risk of bias, indicated that the majority of research design, implementation and analysis did not meet required criteria, and that the research question was too limited to fully address the issue.

## 3. Results

**Figure 1:**
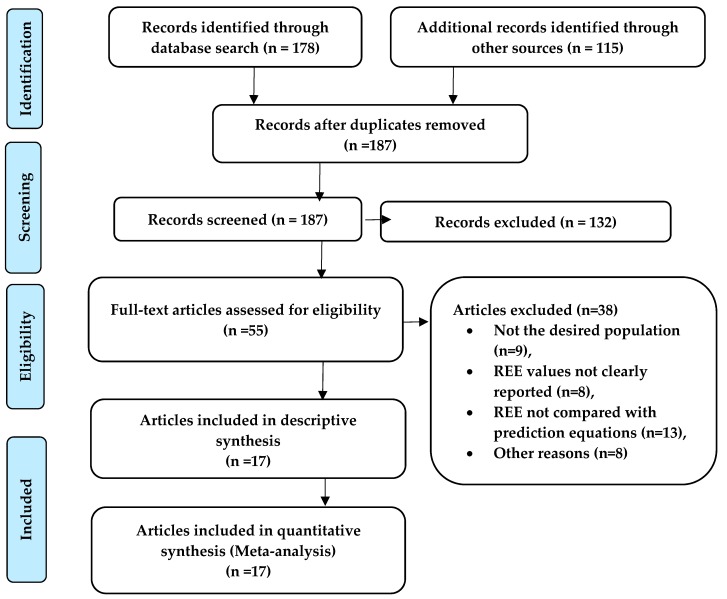
Flow of studies through the selection process.

The literature search identified 187 articles. After applying our predefined initial exclusion criteria and removing duplicates according to search strategies, 55 articles were selected for full-text review. Of these, 38 full-text articles were excluded, leaving 17 for analysis, see Figure 1 for Study flow.

### 3.1. Characteristics of Included Studies

A total of 1883 cirrhotic patients were analyzed across the 17 studies (two separately reporting data in men and women). The cohort’s weighted mean (standard deviation, SD) age was 53 ± 7 years, 65% were men. MELD scores were only reported in six studies with a mean value of 15 (±4). Hepatitis C was the main etiology of liver cirrhosis in 42% of patients, followed by alcohol origin in 33%. CP grade and/or score were reported in 13 studies with 43% CP-A, 39% CP-B and 18% CP-C, respectively. The weighted mean (SD) BMI was 22 (±3) kg/m^2^ across studies. Thirteen studies took into account dry-weight. Seven of these studies either excluded patients with ascites or estimated dry-weight using a variety of techniques. One study [28] used the post-paracentesis weight. Two studies [21,29] mentioned that the dry weight was calculated by deducting an estimated weight for ascites and/or limb edema. Three studies [30,31,32] excluded patients with clinically detectable ascites or peripheral edema of moderate to severe grade. One study [33] used the DC-320 body composition meter to estimate a patient’s dry weight. Demographic characteristics and study designs of included studies are presented in Table 1.

### 3.2. Study Quality

The overall quality rating showed that the majority of studies were categorized as having a low risk of bias, with twelve studies having a low risk of bias, three studies assigned a moderate rating, and two studies having a high risk of bias. The overall quality of the included studies is reported in Table 2.

### 3.3. Energy Expenditure Assessment

- ***Measured REE (IC assessment):*** All 17 studies used IC by metabolic cart for measuring a patient’s energy needs. The most common IC instruments applied in these studies were respiratory gas analyzer, metabolic cart, open-circuit calorimeter and Vmax. Eight studies used the Deltatrac Metabolic Monitor and four studies the MedGraphics IC. As per accepted guidelines there are several steps required for carrying out IC, the majority of studies (61%) missing more than one step in the process.
- ***Predicted REE:*** Overall, IC was compared with 10 different predictive equations: HB (*n* = 14 studies) [21,28,29,30,31,32,33,35,36,40,41,42,43,44], Mifflin-St Jeor (*n* = 2 studies) [21,35], regression equation based on fat-free mass (FFM-based equation) (*n* = 2 studies) [34,37,38,39], Schofield equation [21], Owen equation (FFM-based equation) [21], Cunningham equation (FFM-based equation) [21], Muller equation (FFM-based equation) [21], body surface area (BSA)-based equation [32], and an equation based on Japanese Dietary Reference Intakes (DRI) [33].
- ***Discrepancy between mREE and pREE:*** The mean value of mREE and pREE and the percentage difference identified in each study are presented in Table 3.

**Figure 2:**
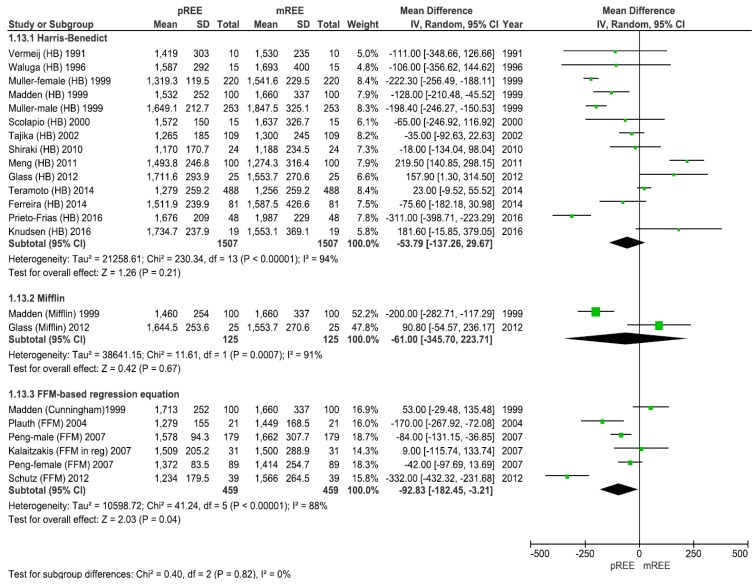
Forest plot of comparison: measured Resting Energy Expenditure (mREE) vs. predicted REE (pREE)—predictive equations. The mean difference between the mREE and pREE was lowest for the HB equation with a non-significant mean difference (*p* = 0.21). The FFM-based equation significantly underestimated the caloric requirements (*p* = 0.04). Abbreviations in parenthesis show the predictive equations employed by each study (i.e., FFM using the individual fat-free mass; HB Harris–Benedict).

When grouping data across studies, 83% of the reviewed predictive equations underestimated the energy requirements of patients with cirrhosis by up to 21% of the mREE. Overestimation was less likely, with 28% of predictive equations overestimating energy requirements within up to 17% of the mREE. We separately analyzed the differences between the mREE and pREE across three equations; the HB equation was the most commonly evaluated equation, used in 14 studies. After HB, the FFM-based equation was evaluated in five studies, and the Mifflin equation in two studies. Although evaluated in a small number of studies, the mean difference between the mREE and pREE was lowest for the HB equation with a non-significant mean difference of −53.8 (95% CI: −137.3, 29.7) kcal/d, *p* = 0.21. The second lowest was the Mifflin equation with a non-significant mean difference of −61 (95% CI: −345.7, 223.7) kcal/d, *p* = 0.67 (Figure 2). The FFM-based equation significantly underestimated the caloric requirements (mean difference of −92.3 (95% CI: −182.4, −3.21) kcal/d, *p* = 0.04), see Figure 2. 

**Subgroup Analysis:** Although multiple subgroup analyses were planned, there was sufficient data to perform analyses only by sex, hospitalization status, study origin, quality rating (low/moderate vs. high risk of bias) and whether dry weight was accounted for (dry vs. no dry weight) for predicting REE. Notably, none of the subgroup analyses were able to completely resolve the heterogeneity seen across studies.

**Figure 3:**
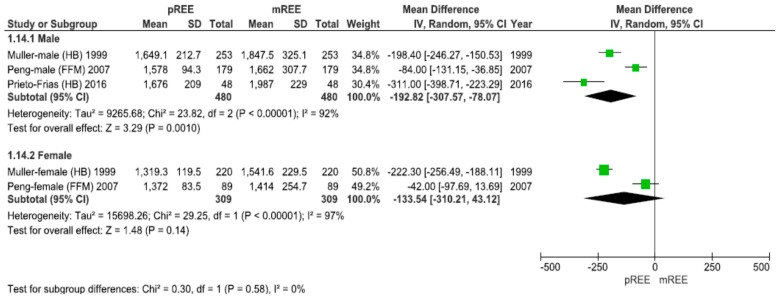
Forest plot of comparison: Measured Resting Energy Expenditure (mREE) vs. predicted REE (pREE)—subgroup: Sex. The aggregated data supports a greater underestimation of energy requirements in male patients versus female patients (*p* = 0.001). Abbreviations in parenthesis show the predictive equations employed by each study (i.e., FFM using the individual fat-free mass; HB Harris–Benedict).

As demonstrated in Figure 3, the aggregated data supports a greater underestimation of energy needs in male patients −192.8 (95% CI: −307.6, −78.1) kcal/d, *p* = 0.001 versus female patients −133.5 (95% CI: −310.2, 43.1) kcal/d, *p* = 0.14. 

**Figure 4:**
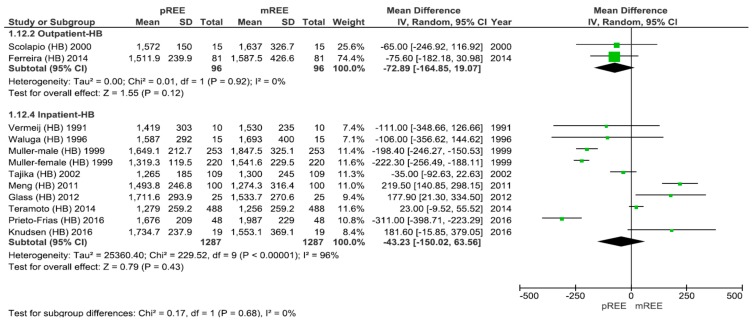
Forest plot of comparison: measured Resting Energy Expenditure (mREE) vs. predicted REE (pREE)—subgroup: Hospitalization status—for the HB equation. There was no statistically significant difference between the value of pREE and mREE in outpatients versus inpatients (*p* = 0.68). **Abbreviation:** HB Harris–Benedict.

Data subdivided by hospitalization status (for the studies utilizing the HB equation) are presented in Figure 4, supporting no statistically significant difference between the value of pREE and mREE in outpatients (mean difference of −72.9; 95% CI: −164.8, 19.1) kcal/d, *p* = 0.12 versus inpatients (mean difference of −43.2; 95% CI: −150, 63.5) kcal/d, *p* = 0.43. 

In a subgrouping of data by study origin (Asian origin studies vs. Western origin studies), Asian studies showed a non-significant tendency to overestimate calorie requirement (mean difference of 47.37; 95% CI: −48.8, 143.4) kcal/d, *p* = 0.33, while in Western studies, the predictive equations significantly underestimated REE in cirrhotic patients (mean difference of −116.2; 95% CI: −175.1, −57.4) kcal/d, *p* = 0.001 (graphical data not shown). 

Subgroup analysis of the evaluated studies by their quality rating showed a significant difference between mREE and pREE within the studies with a low risk of bias. These studies significantly underestimated the energy needs in cirrhotic patients (mean difference of −91.85; 95% CI: −165.12, −18.58) kcal/d, *p* < 0.001, while the underestimation was not significant in studies with a moderate and high risk of bias (mean difference of −14.77; 95% CI: −180.11, 150.57) kcal/d, *p* = 0.86 (graphical data not shown). 

The last subgrouping of data divided studies by whether they accounted for dry weight (or excluded patients with ascites) versus if this was not mentioned (dry vs. no dry weight). This subgrouping did not show any significant difference between the value of mREE and pREE either in the studies reporting dry weight (mean difference of −63.47; 95% CI: −130.13, 3.19) kcal/d, *p* = 0.06, or in those that did not mention or account for it (mean difference of −87.77; 95% CI: −196.65, 21.1) kcal/d, *p* = 0.11 (graphical data not shown). 

## 4. Discussion

Our findings confirm the significant inconsistencies between mREE and pREE in patients with cirrhosis. Notably, only 45% of predictive equations estimated energy requirements to within 90–110% of mREE using IC. Eighty-three percent of predictive equations underestimated and 28% overestimated energy needs by ±10%. The value of ±10% has been a commonly utilized method to indicate an acceptable range of error when comparing mREE and pREE [45,46,47].

When grouping data across the studies using the same predictive equation, the magnitude of the discrepancy between mREE and pREE ranged from an underestimate of 54 to 93 kcal/d across equations. Although this appears to be quite modest when considered in the context of an entire day’s energy requirements, this result must be interpreted with caution. Notably, as demonstrated in Figure 2, using the HB equation, there was significant heterogeneity (I^2^ = 94%) with wide variations in individual comparative data, with several studies demonstrating large discrepancies between the mREE and pREE [28,30,42]. For example, the 95% limits of agreement for predictive values ranged from an underestimate of 399 kcal/day in the work of Prieto-Frias et al. [30] to an overestimate of 379 kcal/day in the work by Knusden et al. [28]. This difference is substantial.

Across equations, the calculated discrepancy was numerically very close. As shown in Figure 2, the HB emerged as having the lowest discrepancy between the pREE and mREE in subgroup analyses. The choice of HB is in keeping with a recommendation by Plauth et al. from the 1997 European Society for Parenteral and Enteral Nutrition (ESPEN) guidelines for nutrition in liver disease and transplantation where it was suggested that the HB equation should be applied for estimating REE in patients with cirrhosis when the IC was not available in a clinical setting [48]. 

The data generated for FFM-based equations is of interest given the prognostic value and prevalence of sarcopenia in cirrhosis. In the current meta-analysis, five studies utilized FFM-based equations (Peng male and female data evaluated separately), see Figure 2. Two of these studies calculated FFM by skinfold anthropometry [21,38], one used prompt gamma in vivo neutron activation analysis (IVNAA) [37], and two studies used bioelectrical impedance analysis (BIA) [34,39]. Although there are many tools for the evaluation of FFM, the emerging gold standard in the cirrhosis literature has been cross-sectional imaging-based muscle mass evaluation by computed tomography or magnetic resonance imaging [49]. To date, we are unaware of cirrhosis studies evaluating whether the measurement of FFM by cross-sectional imaging can improve the accuracy of pREE and lead to a more accurate estimation of the energy requirements [50,51,52]. FFM equations do, of course, carry with them the complexity of requiring a FFM measurement and in certain clinical populations, including patients with obesity, it remains controversial as to whether these equations even improve the accuracy of pREE [47,53,54,55,56]. Further study is warranted in this population.

We attempted to explain the between-study heterogeneity (i.e., identify what made some studies more likely to have a larger discrepancy between the mREE and the pREE than others). Of the subgroup analyses we had planned prehoc, there was insufficient data to carry out the ones based on BMI, etiology of liver disease or liver disease severity. Even in the analyses that were possible, a major limiting factor was that all studies did not provide data for the different subgroups of interest and, therefore, the applicable sample was small for some subgroups. Moreover, even after subgrouping, the overall heterogeneity was not resolved. In the context of these limitations, when the available data were pooled, there was a signal that the underestimation was significantly greater (i) in male patients (data from three studies) and (ii) in studies carried out in the West (versus Asia) (data from 13 studies). As a possible unifying explanation, one could hypothesize that male patients and patients from the West may have an increased fat-free mass as compared to their female and Asian counterparts. Fat-free mass is known to be an important predictor of resting energy expenditure; the resting metabolic rate of adipose tissue is estimated at 4.5 kcal/kg/d as compared to 13 kcal/kg/d for skeletal muscle [23]. Underestimation of fat-free mass by the predictive equations may have led to an underestimation of the energy prescription. The inability of our meta-analysis to identify factors to explain the observed discrepancies between mREE and pREE was also encountered by Madden et al. [21] who evaluated the impact of multiple factors including sex, degree of hepatic decompensation, nutritional status, fluid retention or etiology of liver disease [21]. 

The challenges that we have seen regarding the inaccuracy of predictive equations in our cirrhosis population also apply to other clinical populations, particularly in those patients who are hospitalized, obese or critically ill [57,58,59,60,61]. Although it is challenging to directly compare the accuracy of the equations in cirrhosis with other chronic diseases, many studies have demonstrated substantial inaccuracies. For example, in a recent systematic review, the Harris–Benedict equation prediction was accurate in only 38% to 64% of obese people [62]. Across 513 patients (a mix of obese, inpatients and outpatients), the accuracy of predictive equations was only 8–49%. A systematic review of mechanically ventilated critically ill patients reported underestimation in 38% and overestimation in 12% of patients when calculated using prediction equations [60]. The HB equation in pre-dialytic and dialytic patients with chronic kidney disease has also demonstrated poor agreement with the IC measurement with an accuracy of only 18% [63]. In patients with cirrhosis, it is possible that further evaluation with either prospective studies or individual patient meta-analysis of the existing data will help us to determine in which patients equations are appropriate and which patients will be better served by IC. 

As is commonly carried out in clinical practice, an alternative to the use of predictive equations or IC is energy prescription based on kcal/kg body weight per day based formulas [64]. In our experience, this use of universal kcal/kg body weight equations is common clinical practice, but as supported by evidence in other populations [57], as a trade-off to its simplicity, this practice promotes over-feeding in many patients, particularly the overweight and obese. As we only had mean body weight data available to us in this aggregate meta-analysis, and limited dry-weight estimates, it was not possible to compare the individual values derived by the pREE or mREE to individual weight-based energy requirement estimates. The degree of concordance between these two approaches remains particularly relevant to answer, especially in light of the obesity epidemic [65,66]. Notably, any comparisons will need to take into account the use of the ideal body weight. Ideal body weight is derived from the patient’s height and can be used to estimate the patient’s energy requirement. This particular approach to mitigate the over-prescription of calories using weight-based formulas is recommended by associations such as the International Society for Hepatic Encephalopathy and Nitrogen Metabolism (ISHEN) [67].

There are several noted limitations of the present review, including that the search strategy was restricted to English language studies, which may have led to search bias, and that the review was limited to adult participants. Importantly, although limited to English language studies, there was still broad global representation including countries from North America, Europe, Asia and South America. Additionally, although many authors have investigated the comparison between pREE and mREE in patients with cirrhosis, several studies failed to report pREE values and only reported the percentage of pREE compared to mREE, or compared the two methods of REE measurements—these studies were, therefore, not able to be included in this systematic review. Lastly, there were several subgroup variables with missing data, including data related to liver disease severity. Moreover, studies did not present the discrepancies by subgroup, resulting in an incomplete evaluation of the causes of heterogeneity present within the analysis. Notably, only 54% of studies included in the review mentioned whether they corrected the body weight for excess volume overload (or excluded patients with ascites from the analysis). Although not significant on subgroup analysis, (since we relied on whether it was mentioned in the paper), it was possible that we may not have identified all studies that utilized the dry weight. This discrepancy may have accounted for some of the noted heterogeneity and brings to light the potential for an even greater extent of underestimation of the REE using equations if “water weight” is not accounted for. For example, a 110 kg patient who has 15 kg of ascites and edema is only 95 kg, a factor of 95 vs. 110 in the predictive equations could be quite a large value. Despite these limitations, the study strengths comprise a unique and comprehensive review of the literature using strict inclusion criteria and exclusion criteria.

## 5. Conclusions

This systematic review delineates the significant discrepancies that exist between estimates of frequently used predictive equations and IC measurements in patients with cirrhosis, and their tendency to err towards underestimation of energy needs. Subgroup analyses suggesting a greater underestimation in males and in Western populations require confirmation with larger numbers of patients. We also require future studies with the capacity to subgroup patients by additional factors such as liver disease severity, obesity and acute illness. This may allow identification of those patients with the largest differences between mREE and pREE in whom predictive equations are insufficient and IC is required as a first-line measurement tool. 

How do these results apply to clinical practice? The data from this meta-analysis support the use of IC if available, and the awareness that predictive equations are likely to underestimate needs. Until further data is available, our results support the addition of at least 54 kcal to the pREE calculated by the HB equation in practice. 

In the research realm, these data highlight the need for additional exploration including the assessment of FFM-based predictive equations developed using cross-sectional imaging, validation of the more practical handheld IC and comparison of IC- and predictive-equation-based energy requirements to ESPEN-based kcal/kg body weight guideline recommendations. Using the information available to us we were unable to develop a novel equation for the prediction of calorie requirements. This is also a topic for future studies. All this information will help to guide us forward in our goal to more accurately identifying the energy needs of our patients with cirrhosis. 

## Figures and Tables

**Table 1:** Demographic Characteristics of the included studies.

Author, Year; Country	Cirrhotic Patient Population, Age ^†^	Clinical Setting	BMI [kg/m^2^] ^†^	Etiology of Liver Cirrhosis [%]				Severity of Liver Disease				
				Alcohol	Viral	NAFLD/Cryptogenic	Other ^‡^	CP Grade [%]			CP Score ^†^	MELD Score ^†^
								A	B	C		
Prieto-Frias et al., 2016; Spain [30]	*n* = 48 cirrhosis (all male); age 54 ± 9 yr	Inpatient	28.9 ± 4.3	62.5	10.4 (HBV) 20.9 (HCV)		6.2	31.3	45.8	22.9	-	15 ± 5
Knudsen et al., 2016; Denmark [28]	*n* = 19 cirrhosis (M 16; F 3); mean age 63 ± 8.2 yr	Inpatients with tense ascites	26 ± 4.4	68.4	5.3 (HCV) 21 (HCV + alcohol)	5.3	0	0	42	58	-	13 ± 3.7
Ferreira et al., 2014; Brazil [29]	*n* = 81 cirrhosis (M 59; F 22); mean age 52 ± 8 yr	Outpatient	-	38	9 (HBV) 27 (HCV)	14.8	11.2	25	52	23	-	16 ± 3
Teramoto et al., 2014; Japan [33]	*n* = 488 cirrhosis (M 361; F 127); age 60 ± 0.5 yr	Inpatient	22 ± 0.1	5.5	28.3(HBV), 48.8 (HCV), 2.9 (HCV + HBV)		14.5	62	32	6	-	-
Schutz et al., 2012; Germany [34]	*n* = 39 cirrhosis (M 27; F 12); mean age 59.2 ± 11.5 yr	Inpatient	23.9 ± 3.8	41	7.7	17.9	33.4	0	64	36	-	-
Glass et al., 2012; United States [35]	*n* = 25 cirrhosis (M 17; F 8); age 56.6 ± 8 yr	Inpatient	29.6 ± 7.7	16	36	16	32	-	-	-	9 ± 1.5	17.4 ± 4.4
Meng et al., 2011; China [36]	*n* = 100 cirrhosis (M 75; F 25); age 48.39 ± 5.6 yr	Inpatient	23.8 ± 3.68	0	100 (HBV)	0	0	-	-	-	10.56 ± 1.05	15.64 ± 3.7
Shiraki et al., 2010; Japan [31]	*n* = 24 cirrhosis (M 16; F 8); age 65 ± 6 yr	unclear	21.3 ± 2.4	0	100 (HCV)	0	0	37.5	37.5	25	-	-
Peng et al., 2007; New Zealand [37]	*n* = 268 cirrhosis (M 179; F 89); age 50.1 ± 9.8 yr	unclear	26.9 ± 0.3	16	56		28	34	35	30	-	-
Kalaitzakis et al., 2007; Sweden [38]	*n* = 31 cirrhosis (M 18; F 13); mean age 57 ± 8.9 yr	Outpatient	26.3 ± 3.7	42	16	19	23	36	48	16	8 ± 2.2	11± 3.7
Plauth et al., 2004; Germany [39]	*n* = 21 cirrhosis (M 13; F 8); mean age 60 ± 8 yr	Inpatient	22.3 ± 4.25	90.5	0	0	9.5	-	-	-	-	-
Tajika et al., 2002; Japan [40]	*n* = 109 cirrhosis (M 56; F 53); age 61.2 ± 9 yr	Inpatient	23 ± 3.3	0	11 (HBV) 88 (HCV)	0	1	24	57	19	-	-
Scolapio et al., 2000; United States [41]	*n* = 15 cirrhosis (M 7; F 8); mean age 52 ± 8.5 yr	Outpatient	27.7 ± 7.3	7	33 (HCV)	20	40	40	47	13	-	-
Madden et al., 1999; UK [21]	*n* = 100 cirrhosis (M 56; F 44); mean age 48.5 ± 7.5 yr	In- and outpatients	23.4 ± 4.08	72	14	2	12	32	29	39	-	-
Muller et al., 1999; Germany [42]	*n* = 473 cirrhosis (M 253; F 220); age 44.2 ± 12.6 yr	Inpatient	-	0	40	0.6	59.4	33.9	52.1	14	-	-
Waluga et al., 1996; Poland [32]	*n* = 15 cirrhosis (M 10; F 5); age 37.7 ± 9.9 yr	Inpatient	24.7 ± 4.2	0	87	13	0	-	-	-	-	-
Vermeij et al., 1991; Poland [43]	*n* = 10 cirrhosis (M 5; F 5); age 48 ± 14 yr	Inpatient	-	40	40	0	20	40	10	50	-	-

^†^ Values are presented in Mean ± SD. ^‡^ Other etiologies includes autoimmune disease, cryptogenic, hemochromatosis, primary biliary cirrhosis, Wilson’s disease, Budd–Chiari syndrome, Crigler Najjar syndrome, etc. **Abbreviations:** BMI body mass index; CP Child-Pugh; HBV hepatitis B virus; HCV hepatitis C virus; MELD model for end-stage liver disease; NAFLD non-alcoholic fatty liver disease.

**Table 2:** Individual study risk of bias in accordance with PRISMA Statement.

Study	Risk of Bias					
	Selection	Performance	Attrition	Detection	Statistical Analysis and Reporting	Overall Study Risk of Bias
Prieto-Frias et al. [30]	high	moderate	low	low	low	Moderate
Knudsen et al. [28]	low	low	low	moderate	low	Low
Ferreira et al. [29]	moderate	low	low	low	moderate	Low
Teramoto et al. [33]	moderate	low	low	low	low	Low
Schutz et al. [34]	moderate	high	low	moderate	low	High
Glass et al. [35]	low	high	moderate	moderate	low	High
Meng et al. [36]	low	moderate	low	moderate	low	Low
Shiraki et al. [31]	low	low	low	moderate	moderate	Low
Peng et al. [37]	low	moderate	low	moderate	low	Low
Kalaitzakis et al. [38]	low	moderate	low	moderate	moderate	Moderate
Plauth et al. [39]	moderate	moderate	low	low	low	Low
Tajika et al. [40]	low	high	moderate	moderate	low	Moderate
Scolapio et al. [41]	low	low	low	moderate	moderate	Low
Madden et al. [21]	low	low	low	low	low	Low
Muller et al. [42]	low	low	low	low	low	Low
Waluga et al. [32]	moderate	low	low	low	moderate	Low
Vermeij et al. [43]	low	low	low	low	low	Low

**Table 3:** Description of the studies included in the meta-analyses.

Study	Study Design	Number of Subjects (Cirrhosis)	Intervention		Outcomes		
			Calorimetry Method Criteria Met (Missing Techniques)	Equation (Weight ^¶^) Used to Predict REE	mREE ^†^ (kcal/day)	pREE ^†^ (kcal/day)	% Difference ^‡^ (-) Underestimate (+) Overestimate
Prieto-Frias et al. [30]	Prospective controlled study;	48 (M)	Yes	HB (dry wt.)	1987 ± 229	1676 ± 209	−15.65
Knudsen et al. [28]	Prospective study;	19 (M 16; F 3)	No (fasting, prior resting, temperature-controlled room)	HB (dry wt.)	1553.1 ± 369.1	1734.7 ± 237.9	11.69
Ferreira et al. ^§^ [29]	Randomized controlled trial;	81 (M 59; F 22)	Yes	HB (dry wt.)	1587.5 ± 426.6	1511.9 ± 239.9	−4.76
Teramoto et al. [33]	Cross-sectional study;	488 (M 361; F 127)	No (temperature-controlled room)	HB, equation based on DRI for Japanese (dry wt.)	1256 ± 247.39	1279 ± 247.39 (HB) 1254 ± 247.39 (Japanese DRI)	1.83 (HB) −0.16 (Japanese DRI)
Schutz et al. [34]	Cross-sectional study;	39 (M 27; F 12)	No (length of measurement, calibration, temperature-controlled room)	BCM based-regression equation (-)	1566 ± 264.5	1234 ± 179.5	−21.2
Glass et al. [35]	Prospective controlled study;	25 (M 17; F 8)	No (prior resting, fasting, temperature-controlled room	HB, Mifflin (-)	1553.7 ± 270.6	1711.6 ± 293.9 (HB) 1644.5 ± 253.6 (Mifflin)	10.16 (HB) 5.84 (Mifflin)
Meng et al. [36]	Retrospective cohort;	100 (M 75; F 25)	No (length of measurement, steady state)	HB (-)	1274.27 ± 316.36	1493.80 ± 246.80	17.23
Shiraki et al. [31]	Prospective controlled study;	24 (M 16; F 8)	No (fasting, length of measurement, temperature-controlled room)	HB (dry wt.)	1188 ± 234.5	1170 ± 170.75	−1.51
Peng et al. [37]	Cross-sectional study;	268 (M 179; F 89)	No (fasting, steady state, calibration, length of measurement, temperature-controlled room)	Regression equation based on FFM (dry wt.)	M: 1662 ± 23 F: 1414 ± 27	M: 1578 ± 10 F: 1372 ± 15	M: −5.05 F: −2.97
Kalaitzakis et al. [38]	Cross-sectional study;	31 (M 18; F 13)	No (calibration, steady state, prior resting, length of measurement, temperature-controlled room)	Regression equation based on FFM (dry wt.)	1500 ± 288.89	1509 ± 205.18	0.6
Plauth et al. [39]	Prospective study;	21 (M 13; F 8)	No (fasting, calibration, steady state, prior resting, length of measurement, temperature-controlled room)	BCM-based regression equations (-)	1449 ± 168.5	1279 ± 155	−11.73
Tajika et al. [40]	Prospective, consecutive-entry study;	109 (M 56; F 53)	No (fasting, calibration, steady state, prior resting, length of measurement, temperature-controlled room)	HB (-)	1300 ± 245	1265 ± 185	−2.69
Scolapio et al. [41]	Cross-sectional study;	15 (M 7; F 8)	No (prior resting, length of measurement)	HB (-)	1637 ± 326.75	1572 ± 150	−3.97
Madden et al. [21]	Cross-sectional study;	100 (M 56; F 44)	No (fasting)	HB, Schofield, Mifflin, Cunningham, Owen, Muller equations (dry wt.)	1660 ± 337	1532 ± 252 (HB) 1575 ± 254 (Schofield) 1460 ± 254 (Mifflin) 1713 ± 252 (Cunningham) 1521 ± 281 (Owen) 1783 ± 204 (Muller)	−7.71 (HB) −5.12 (Schofield) −12.05 (Mifflin) 3.19 (Cunningham) −8.37 (Owen) 7.4 (Muller)
Muller et al. [42]	Cross-sectional study;	473 (M 253; F 220)	No (temperature-controlled room)	HB (-)	M: 1847.51 ± 325.05 F: 1541.59 ± 229.45	M: 1649.14 ± 212.72 F: 1319.31 ± 119.5	M: −10.74 F: −14.42
Waluga et al. [32]	Cross-sectional study;	15 (M 10; F 5)	Yes	HB, BSA-based regression equation (dry wt.)	1693 ± 400	1571 ± 291 (HB) 1587 ± 292 (BIA)	−7.21
Vermeij et al. [43]	Prospective controlled study;	10 (M 5; F 5)	No (temperature-controlled room)	HB(-)	1530 ± 235	1419 ± 303	−7.25

^†^ Values are presented in Mean ± SD. ^‡^ % Difference = (pREE-mREE/mREE)*100. ^¶^ Weight used in predictive equations: (1) dry weight (directly mentioned that the dry weight was measured or patients with ascites were excluded from the study, so the scale weight is equal to the estimated dry weight), (2) (-) not mentioned which weight (dry or no dry wt.) used for calculating pREE in patients with ascites. ^§^ Only the baseline data was used for this meta-analysis. **Abbreviations:** BCM body cell mass; BSA body surface area; dry wt. dry weight; DRI Dietary Reference Intakes; F Female; FFM fat-free mass; HB Harris–Benedict; IC Indirect Calorimetry; M Male; mREE measured resting energy expenditure using indirect calorimetry; REE Resting Energy Expenditure; pREE predicted resting energy expenditure using predictive equations.

## Supplementary Materials

The following are available online at http://www.mdpi.com/2072-6643/11/2/334/s1, Table S1: Search criteria, Table S2: Research design areas, Table S3: Predictive equations used to estimate resting energy expenditure.
