# Supplementary material for: Are Predictive Energy Expenditure Equations Accurate in Cirrhosis?
Source: Nutrients. 2019 Feb 4;11(2):334. doi: 10.3390/nu11020334 (PMC6412603; doi:10.3390/nu11020334)
Supplement: Supplementary file 1 [file nutrients-11-00334-s001.pdf]

Supplementary data

1. **Table S1.** Search criteria

MEDLINE and EMBASE search criteria

1. exp direct calorimetry/ or exp calorimetry/ or exp indirect calorimetry/ or Calorimetry.mp.
2. indirect calorimetry.mp. or exp calorimetry/ or exp energy metabolism/ or exp indirect calorimetry/ or exp energy expenditure/ or exp metabolism/
3. exp energy metabolism/ or direct calorimetry.mp. or exp calorimetry/ or exp direct calorimetry/ or exp energy expenditure/
4. resting energy expenditure.mp. or exp calorimetry/ or exp malnutrition/ or exp energy metabolism/ or exp metabolism/ or exp energy expenditure/ or exp resting energy expenditure/ or exp body composition/
5. exp body composition/ or resting metabolic rate.mp. or exp basal metabolic rate/ or exp body weight/ or exp energy expenditure/ or exp obesity/ or exp metabolic rate/ or exp metabolism/ or exp resting metabolic rate/
6. exp oxygen consumption/ or hypermetabolism.mp. or exp metabolism/ or exp hypermetabolism/
7. 1 or 2 or 3 or 4 or 5 or 6 or 7
8. exp liver cirrhosis/
9. exp chronic liver disease/ or End Stage Liver Disease.mp. or exp liver transplantation/ or exp liver cirrhosis/ or exp end stage liver disease/ or exp liver disease/ or exp liver failure/
10. chronic liver disease.mp. or exp liver disease/ or exp chronic liver disease/ or exp hepatitis/ or exp liver cirrhosis/
11. 9 or 10 or 11
12. exp metabolism/ or exp indirect calorimetry/ or exp calorimetry/ or exp energy expenditure/ or exp resting energy expenditure/ or Harris- Benedict.mp. or exp malnutrition/
13. exp basal metabolic rate/ or exp prediction/ or exp resting energy expenditure/ or Mifflin.mp. or exp energy expenditure/
14. exp caloric intake/ or exp resting energy expenditure/ or exp energy expenditure/ or exp calorimetry/ or exp metabolism/ or Schofield.mp. or exp prediction/
15. exp basal metabolic rate/ or exp energy expenditure/ or exp prediction/ or exp calorimetry/ or predictive equation.mp. or exp resting energy expenditure/
16. 13 or 14 or 15 or 16
17. 8 and 12 and 17
18. limit 18 to (human and English language)

Scopus Search

((TITLE-ABS-KEY (calorimetry) OR TITLE-ABS-
KEY (indirect AND calorimetry) OR TITLE ABS-
KEY (resting AND energy AND expenditure) OR TITLE-ABS
KEY (respiratory AND quotient) OR TITLE-ABS-
KEY (hypermetabolism))) AND ((TITLE ABS-
KEY (liver AND cirrhosis) OR TITLE-ABS-KEY (end
stage AND liver AND disease) OR TITLE-ABS-

Supplementary data

KEY (liver AND transplantation) OR TITLE ABS-KEY (chronic AND liver AND disease))) AND ((TITLE-ABS-KEY (harris benedict) OR TITLE-ABS-KEY (mifflin) OR TITLE-ABS-KEY (schofield) OR TITLE-ABS KEY (predictive AND equation)))
**PubMed search – Dec. 15, 2017 was last searched day**
("Calorimetry"[Mesh] OR "indirect calorimetry"[All Fields] OR "resting energy expenditure"[All Fields] OR "respiratory quotient"[All Fields] OR "hypermetabolism"[All Fields) AND ("Liver Cirrhosis"[Mesh] OR "End-stage liver disease"[MeSH] OR "end-stage liver"[All Fields] OR "liver transplantation"[All Fields] OR "chronic liver disease"[All Fields] OR "cirrhosis"[All Fields]) AND ("harris-benedict"[All Fields] OR "mifflin"[All Fields] OR "schofield"[All Fields] OR "predictive equation"[All Fields])AND ("english"[Filter]) AND ("humans"[Filter] OR "men"[All Fields] OR "women"[All Fields])

Supplementary data

Table S2. Research design areas

Research design domains assessed before rating the quality of a study [1,2]**:**

2. Well-defined research question, compared with the review question
3. Free of selection bias (using randomization or consecutive sampling)
4. Histological confirmation of liver cirrhosis
5. Explanation of exclusion criteria and withdrawal method
6. Clear description of measurements
7. Free of outcome reporting bias (indirect calorimetry measurement bias)
   - Machine calibration before each exam
   - Resting for at least 20-30 min prior to the procedure (15-min rest period if the first 5 min are discarded), No exercising for 4 hours prior to the measurement
   - Provision of a temperature-controlled room (22-24℃)
   - Steady status (Maintain a supine position throughout the measurement period)
   - Fasting time (overnight, or 5 to more hours)
   - Measurement length (10 - 30 min period)
8. Appropriate statistical analyses
9. Conclusion supported by findings
10. Free of funding bias

Supplementary data

Table S1. Predictive equations used to estimate resting energy expenditure

HB[3]	**M:** REE = 66.47 + (13.75 × wt) + (500.33 × ht) – (6.76 × age)<br>**F:** REE = 655.10 + (9.56 × wt) + (184.96 × ht) – (4.68 × age)
Mifflin-St Jeor[4]	**M:** REE = (9.99 × wt) + (625 × ht) – (4.92 × age) + 5<br>**F:** REE = (9.99 × wt) + (625 × ht) – (4,92 × age) - 161
Schofield [5]	**M: (18-30 years)** REE = (15.06 × wt) + 692.2<br>**(30-60 years)** REE = (11.48 × wt) + 873.1<br>**(+60 years)** REE = (9.09 × wt) + 587.7<br>**F: (18-30 years)** REE = (14.82 × wt) + 486.6<br>**(30-60 years)** REE = (8.13 × wt) + 845.6<br>**(+60 years)** REE = (9.08 × wt) + 658.5
Owen[6]	REE = 169 + (24.1 × FFM)
Cunningham [7]	REE = 502 + (21.6 × FFM)
Muller [8]	REE = 1052 + (17.08 × FFM) – (4.6 × age)
FFM-based regression equation[9,10]	**(1)** REE = 16.85 × FFM + 725<br><br>**(2)** BCM_{BIA} = FFM × 0.29 × ln (α)<br>**M:** REE = 28.76 × BCM + 727.07<br>**F:** REE = 25.82 × BCM + 784.96
BSA-based regression equation	BSA = 0.0235 × $H^{0.42246}$ × $W^{0.51456}$ (not published the final equation)
Equation based on Japanese DRI[11]	Not published
**Abbreviations:** *HB* Harris-Benedict; *M* Male; *F* Female; *REE* Resting Energy Expenditure; *IC* Indirect Calorimetry; *wt* weight; *ht* height; *FFM* fat free mass; ; *BCM* body cell mass; *BIA* bio-impedance analysis; *BSA* body surface area; *DRI* Dietary Reference Intakes	

References

1. Haugen, H.A.; Chan, L.; Li, F. Indirect Calorimetry: A Practical Guide for Clinicians. Nutrition in Clinical Practice **2007**, *22*, 377-388.
2. Stroup, D.F.; Berlin, J.A.; Morton, S.C.; Olkin, I.; Williamson, G.D.; Rennie, D.; Moher, D.; Becker, B.J.; Sipe, T.A.; Thacker, S.B. Meta-Analysis of Observational Studies in Epidemiology: A Proposal for Reporting. JAMA **2000**, *283*, 2008-2012.
3. Harris, J.A.; Benedict, F.G. A Biometric Study of Human Basal Metabolism. Proc. Natl. Acad. Sci. U. S. A. **1918**, *4*, 370-373.
4. Mifflin, M.D.; St Jeor, S.T.; Hill, L.A.; Scott, B.J.; Daugherty, S.A.; Koh, Y.O. A New Predictive Equation for Resting Energy Expenditure in Healthy Individuals. Am. J. Clin. Nutr. **1990**, *51*, 241-247.
5. Schofield, W.N. Predicting Basal Metabolic Rate, New Standards and Review of Previous Work. Hum. Nutr. Clin. Nutr. **1985**, *39 Suppl 1*, 5-41.
6. Owen, O.E.; Trapp, V.E.; Reichard, G.A.,Jr; Mozzoli, M.A.; Moctezuma, J.; Paul, P.; Skutches, C.L.; Boden, G. Nature and Quantity of Fuels Consumed in Patients with Alcoholic Cirrhosis. J. Clin. Invest. **1983**, *72*, 1821-1832.

Supplementary data

7. Cunningham, J.J. A Reanalysis of the Factors Influencing Basal Metabolic Rate in Normal Adults. Am. J. Clin. Nutr. **1980**, *33*, 2372-2374.
8. Muller, M.J.; Bosy-Westphal, A.; Klaus, S.; Kreymann, G.; Luhrmann, P.M.; Neuhauser-Berthold, M.; Noack, R.; Pirke, K.M.; Platte, P.; Selberg, O. *et al.* World Health Organization Equations have Shortcomings for Predicting Resting Energy Expenditure in Persons from a Modern, Affluent Population: Generation of a New Reference Standard from a Retrospective Analysis of a German Database of Resting Energy Expenditure. Am. J. Clin. Nutr. **2004**, *80*, 1379-1390.
9. Plauth, M.; Schütz, T.; Buckendahl, D.P.; Kreymann, G.; Pirlich, M.; Grüngreiff, S.; Romaniuk, P.; Ertl, S.; Weiß, M.; Lochs, H. Weight Gain After Transjugular Intrahepatic Portosystemic Shunt is Associated with Improvement in Body Composition in Malnourished Patients with Cirrhosis and Hypermetabolism. J. Hepatol. **2004**, *40*, 228-233.
10. Kalaitzakis, E.; Bosaeus, I.; Ohman, L.; Bjornsson, E. Altered Postprandial Glucose, Insulin, Leptin, and Ghrelin in Liver Cirrhosis: Correlations with Energy Intake and Resting Energy Expenditure. Am. J. Clin. Nutr. **2007**, *85*, 808-815.
11. Teramoto, A.; Yamanaka-Okumura, H.; Urano, E.; Nakamura-Kutsuzawa, T.; Sugihara, K.; Katayama, T.; Miyake, H.; Imura, S.; Utsunomiya, T.; Shimada, M. Comparison of Measured and Predicted Energy Expenditure in Patients with Liver Cirrhosis. Asia Pac. J. Clin. Nutr. **2014**, *23*, 197-204.
